# *Prdm6* Is Essential for Cardiovascular Development *In Vivo*

**DOI:** 10.1371/journal.pone.0081833

**Published:** 2013-11-21

**Authors:** Andreas Gewies, Mercedes Castineiras-Vilarino, Uta Ferch, Nina Jährling, Katja Heinrich, Ulrike Hoeckendorf, Gerhard K. H. Przemeck, Matthias Munding, Olaf Groß, Timm Schroeder, Marion Horsch, E. Loraine Karran, Aneela Majid, Stefan Antonowicz, Johannes Beckers, Martin Hrabé de Angelis, Hans-Ulrich Dodt, Christian Peschel, Irmgard Förster, Martin J. S. Dyer, Jürgen Ruland

**Affiliations:** 1 Institut für Klinische Chemie und Pathobiochemie, Klinikum Rechts der Isar, Technische Universität München, Munich, Germany; 2 German Cancer Consortium (DKTK), Heidelberg, Germany; 3 German Cancer Research Center (DKFZ), Heidelberg, Germany; 4 Laboratory of Signaling in the Immune System, Helmholtz Zentrum München, German Research Center for Environmental Health, Neuherberg, Germany; 5 Department of Bioelectronics, Institute of Solid State Electronics, Vienna University of Technology, Vienna, Austria; 6 Center for Brain Research, Section of Bioelectronics, Medical University of Vienna, Vienna, Austria; 7 Department of Neurobiology, University of Oldenburg, Oldenburg, Germany; 8 Institute of Experimental Genetics, Helmholtz Zentrum München, German Research Center for Environmental Health, Neuherberg, Germany; 9 Helmholtz Zentrum München, German Research Center for Environmental Health, Research Unit Stem Cell Dynamics, Neuherberg, Germany; 10 MRC Toxicology Unit and Department of Cancer Studies and Molecular Medicine, University of Leicester, Leicester, United Kingdom; 11 Chair of Experimental Genetics, Technische Universität München, Freising-Weihenstephan, Germany; 12 Department of Internal Medicine III, Klinikum Rechts der Isar, Technische Universität München, Munich, Germany; 13 Institute of Medical Microbiology, Immunology and Hygiene, Technische Universität München, Munich, Germany; 14 Immunology and Environment, Life and Medical Sciences (LIMES) Institute, University of Bonn, Bonn, Germany; 15 German Center for Infection Research (DZIF), partner site München, Munich, Germany; Institute of Neurology (Edinger-Institute), Germany

## Abstract

Members of the PRDM protein family have been shown to play important roles during embryonic development. Previous *in vitro* and *in situ* analyses indicated a function of Prdm6 in cells of the vascular system. To reveal physiological functions of Prdm6, we generated conditional *Prdm6*-deficient mice. Complete deletion of *Prdm6* results in embryonic lethality due to cardiovascular defects associated with aberrations in vascular patterning. However, smooth muscle cells could be regularly differentiated from *Prdm6*-deficient embryonic stem cells and vascular smooth muscle cells were present and proliferated normally in *Prdm6*-deficient embryos. Conditional deletion of *Prdm6* in the smooth muscle cell lineage using a SM22-Cre driver line resulted in perinatal lethality due to hemorrhage in the lungs. We thus identified Prdm6 as a factor that is essential for the physiological control of cardiovascular development.

## Introduction

Prdm6 belongs to the PRDM family of transcriptional repressors which all possess an N-terminal PR domain and C-terminal Krüppel-type zinc finger motifs. While the zinc fingers are responsible for DNA binding, the PR domain is thought to mediate homodimerization and interaction with proteins such as the histone methyl transferase G9a and histone deacetylases [1-3]. Therefore, PRDM proteins are expected to play important roles as histone modifying factors that regulate gene transcription at the chromatin level. The most intensely studied PRDM member PRDM1 (also named BLIMP1) has been shown to mediate methylation of lysine residue 9 of histone 3 [2] and as a transcriptional repressor has been demonstrated to be essential for several physiological processes such as terminal B cell differentiation [4], T cell homeostasis and function [5,6], primordial germ cell formation [7] and regulation of proliferation and differentiation in the sebacious gland [8]. Other members of the PRDM family were also reported to control developmental processes: Prdm5 regulates collagen gene transcription in developing bone [9], Prdm9 defines hotspots of genetic recombination during meiosis [10], Prdm14 was shown to be involved in the maintenance of embryonic stem cells in the mouse [11], and Prdm16 controls the bidirectional switch between skeletal myoblasts and brown fat cells [12]. While PRDM transcription factors control various developmental processes under physiological conditions, aberrant expression of PRDM proteins has been correlated with malignant disease and *PRDM* genes map to chromosomal regions frequently deleted in tumors [13-16]. Moreover, PRDM proteins can be expressed as PR domain-containing full length proteins or as amino-terminally truncated proteins lacking a functional PR domain by usage of an alternative internal promoter. Loss of *PRDM* full length expression or a shift in expression towards the truncated shorter form has been implicated in tumorigenesis [2,15,17-20]. 

Prdm6 was recently characterized as a transcriptional repressor that is expressed and plays a role in the vascular system. Davis and colleagues described Prdm6 as a transcription factor that plays a role in regulating the differentiation and proliferation of smooth muscle cells (SMCs) [3]. Moreover, Prdm6 was described as a factor that controls survival and differentiation of endothelial cells in the vascular system [21]. Furthermore, expression of *Prdm6* has been reported in cells of the developing nervous system [22]. Finally, we identified *PRDM6* to be transcriptionally deregulated and ectopically expressed from the rare, but recurrent chromosomal translocation t(5;14)(q23;q32) in B cell lymphoma patients (manuscript in preparation). To reveal physiological functions of Prdm6 *in vivo*, we generated and analyzed conditional *Prdm6*-deficient mice. We report here that Prdm6 is essential for embryonic development and for vital functions of the cardiovascular system.

## Results

### Generation of a conditional *PRDM6* mutant mouse line

Because the physiological functions of PRDM6 are still largely unknown, we generated a gene-targeted mouse line that allows conditional *Prdm6* ablation using Cre-*loxP* technology [23]. By homologous recombination in murine embryonic stem (ES) cells we flanked exon 3 of *Prdm6* with *loxP* sites (see Materials and Methods and Figure S1 A,B). After injection of ES cells into blastocysts and removal of the neomycin selection cassette via flp-mediated deletion we eventually obtained *Prdm6* ^wt/flox^ and *Prdm6* ^flox/flox^ mice, which were born at expected Mendelian ratios and were phenotypically indistinguishable from their wild type littermates (data not shown). Crossing *Prdm6* ^wt/flox^ mice to Cre deleter mice [24] induced the deletion of the *loxP*-flanked exon 3 sequence in the germ line and resulted in *Prdm6* ^wt/del^ heterozygous mice. Correct homologous recombination events were confirmed by Southern blot analysis (Figure S1 C). Of note, deletion of *Prdm6* exon 3 not only removes the central part of the PR domain but, due to a concomitant frame shift, it also prevents the expression of the complete *Prdm6* reading frame downstream of the PR domain so that no functional protein can be expressed.

### Prdm6 is essential during embryonic development

**Figure 1:**
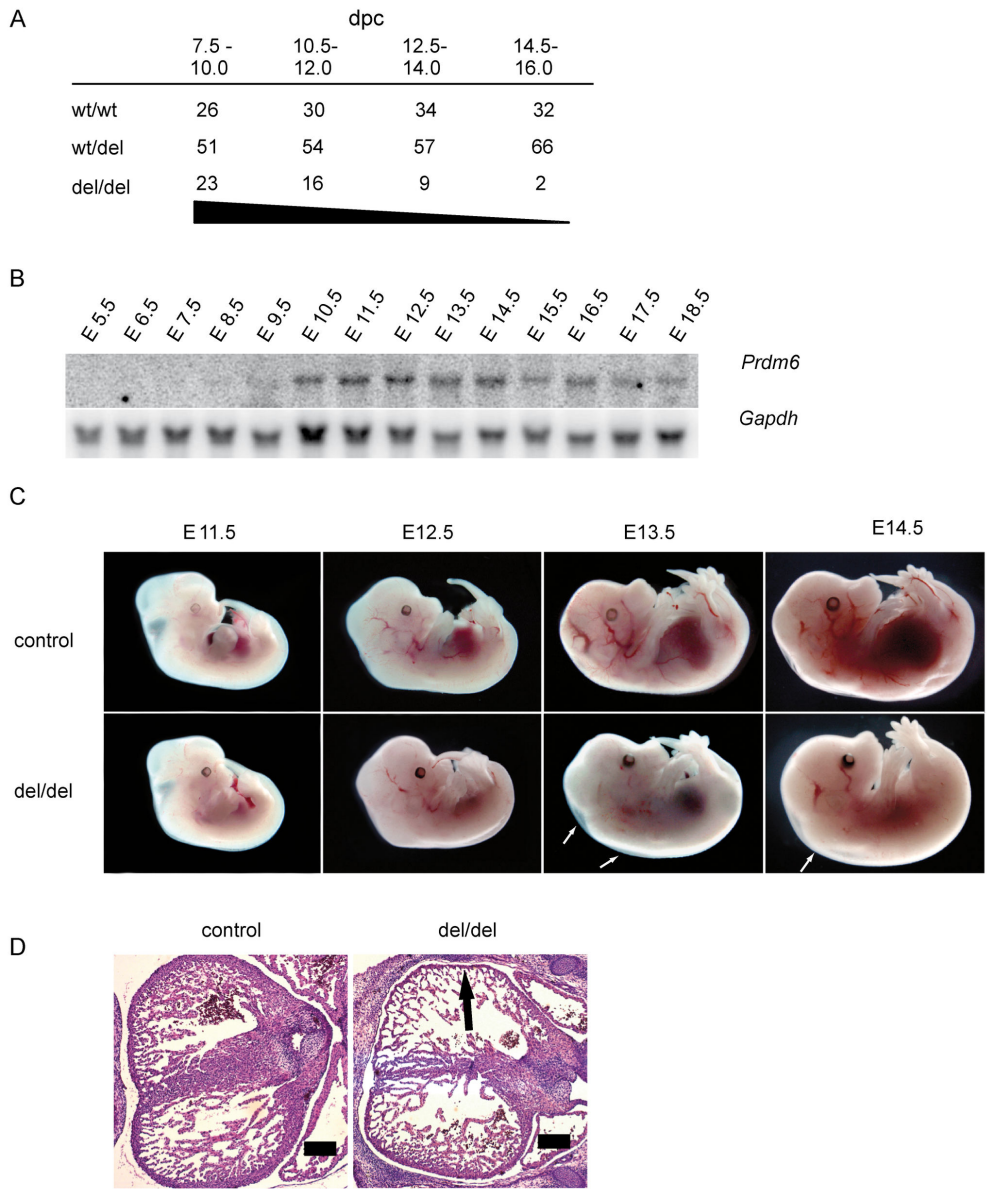
*Prdm6* deficiency results in embryonic lethality. (**A**) *Prdm6* ^wt/del^ mice were intercrossed. Pregnant mice were euthanized and embryos dissected and genotyped at defined developmental stages. The percentages of viable embryos of the respective genotypes at the different stages of embryonic development (dpc = days post coitum) are indicated; wild type *Prdm6* ^wt/wt^ (wt/wt) and heterozygous *Prdm6* ^wt/del^ (wt/del) mice are viable, whereas *Prdm6*-deficient *Prdm6* ^*del/del*^ (del/del) embryos begin to die after E10.0, with no *Prdm6* ^*del/del*^ embryos being found at developmental stages beyond E16.0. (**B**) Northern blot analysis of *Prdm6* expression using total embryonic RNA from different developmental stages from wild type embryos. *Gapdh* expression analysis served as a loading control. (**C**) Representative wild type control and *Prdm6*-deficient embryos (del/del) at the indicated developmental stages. White arrows indicate edematous swelling. (**D**) Transverse heart sections from wild type control and *Prdm6*-deficient embryos were stained with H&E and analyzed by microscopy. The thin myocardium of *Prdm6*-deficient embryos (del/del) is indicated by an arrow. Scale bars correspond to 200 µm.

Heterozygous *Prdm6* ^wt/del^ mice were intercrossed to obtain homozygous *Prdm6*-deficient mutants (*Prdm6* ^del/del^). *Prdm6* ^wt/wt^ and *Prdm6* ^wt/del^ mice were born at the expected Mendelian ratios. In contrast, we did not observe viable homozygous *Prdm6* ^del/del^ offspring, indicating that functional *Prdm6* expression is essential for the viability of mice. Thus, we next performed timed pregnancies and analyzed *Prdm6* ^del/del^ embryos at different developmental stages. Up to E10.0, we observed Mendelian frequencies of morphologically intact *Prdm6* ^del/del^ embryos. However, beyond E10.0 the frequency of viable *Prdm6* ^del/del^ embryos declined significantly whereas *Prdm6* ^wt/wt^ and *Prdm6* ^wt/del^ embryos were present at regular numbers (Figure 1A). RT-PCR analysis confirmed the absence of *Prdm6* exon 3 and therefore the deficiency of functional *Prdm6* mRNA in *Prdm6* ^del/del^ embryos. An alternative *exon2*–*exon4*-spliced *Prdm6* transcript is produced by the disrupted (del) allele in *Prdm6* ^del/del^ and *Prdm6* ^wt/del^ embryos (Figure S1 D). However, since *Prdm6* ^wt/del^ heterozygous mice are born at Mendelian ratios and are viable, healthy and fertile, there is no indication for a dominant gain-of-function of the alternatively spliced *Prdm6* transcript derived from the knockout (del) allele. The onset of embryonic lethality in the *Prdm6* ^del/del^ embryos correlated with the onset of *Prdm6* expression at E10.5 in wild type embryos (Figure 1B), a developmental stage at which the cardiovascular system undergoes critical developmental steps [25]. Macroscopic inspection of *Prdm6* ^del/del^ embryos revealed that at E12.5 they eventually displayed pale and edematous bodies, implying cardiovascular insufficiency (Figure 1C). Analysis of the cardiac architecture of the *Prdm6* ^del/del^ embryos via histological H&E staining revealed a thinning of the myocardial walls, indicating primary or secondary heart failure (Figure 1D). 

### Prdm6 affects vascular patterning

**Figure 2:**
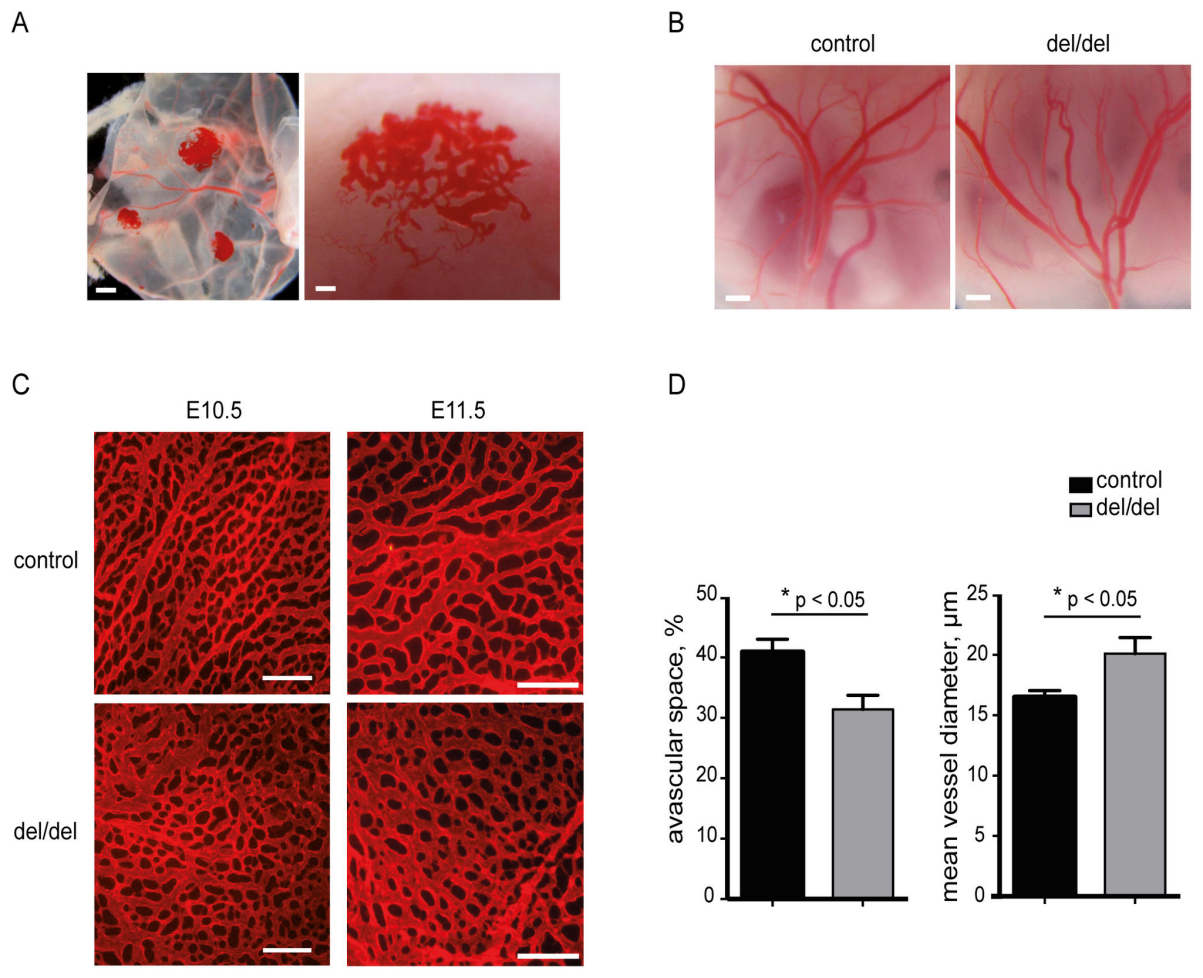
Prdm6 affects angiogenic patterning. (**A**) Unusual clusters of densely growing vessel structures on yolk sacs of *Prdm6*-deficient embryos, as observed under a stereomicroscope. Scale bars correspond to 1 mm (left panel) or 100 µm (right panel). (**B**) Large vessels in the yolk sacs of E12.5 control and *Prdm6*-deficient (del/del) embryos under a stereomicroscope. Scale bars correspond to 500 µm. (**C**) Visualization of E10.5 (left panels) and E11.5 (right panels) yolk sac microvascular systems *via* immunofluorescent staining with an anti-CD31 primary antibody and a Cy3-conjugated secondary antibody. Scale bars correspond to 200 µm. (**D**) Quantitative morphometric analysis of the yolk sac vasculature as shown in (C). Avascular space and mean vessel diameters of yolk sacs at E10.5 – E 11.5 are shown as mean ± SEM, n=6. More details about this analysis are given in Figure S2.

During the isolation of *Prdm6* ^del/del^ embryos from the deciduae, we repeatedly observed vascular malformations exclusively on the yolk sacs of *Prdm6* ^del/del^ embryos at stage ≥E13.5 (Figure 2A, left panel). Higher magnifications revealed that these malformations were composed of clusters of densely growing and partially dilated blood microvessels (Figure 2A, right panel). However, large vessel vascularization was present in the yolk sacs of *Prdm6*-deficient embryos, indicating regular overall vasculogenesis (Figure 2B). To investigate the role of Prdm6 in the development of the small blood vessel architecture in structural detail, we stained vascular endothelial cells with an anti-CD31 (anti-PECAM1) antibody and visualized the yolk sac vascular system via fluorescence microscopy. The yolk sacs of wild type control embryos displayed an organized vascular network with a hierarchy between vessels of higher and lower orders at the developmental stages E10.5 and E11.5 (Figure 2C, upper panels). Also in *Prdm6* ^del/del^ yolk sacs a small vessel network was present (Figure 2C, lower panels). However, visual inspection suggested subtle differences in the patterning of the vascular network of *Prdm6* ^del/del^ yolk sacs compared to wild type control yolk sacs (Figure 2C). Indeed, quantitative analysis revealed that the small vessel network of *Prdm6* ^del/del^ yolk sacs contains significantly less avascular space and exhibits a significant increase in the mean vessel diameter (Figure 2D and Figure S2). These findings indicate that Prdm6 function is involved in vascular patterning during embryonic development. 

### Normal differentiation and proliferation of *Prdm6-*deficient smooth muscle cells

**Figure 3:**
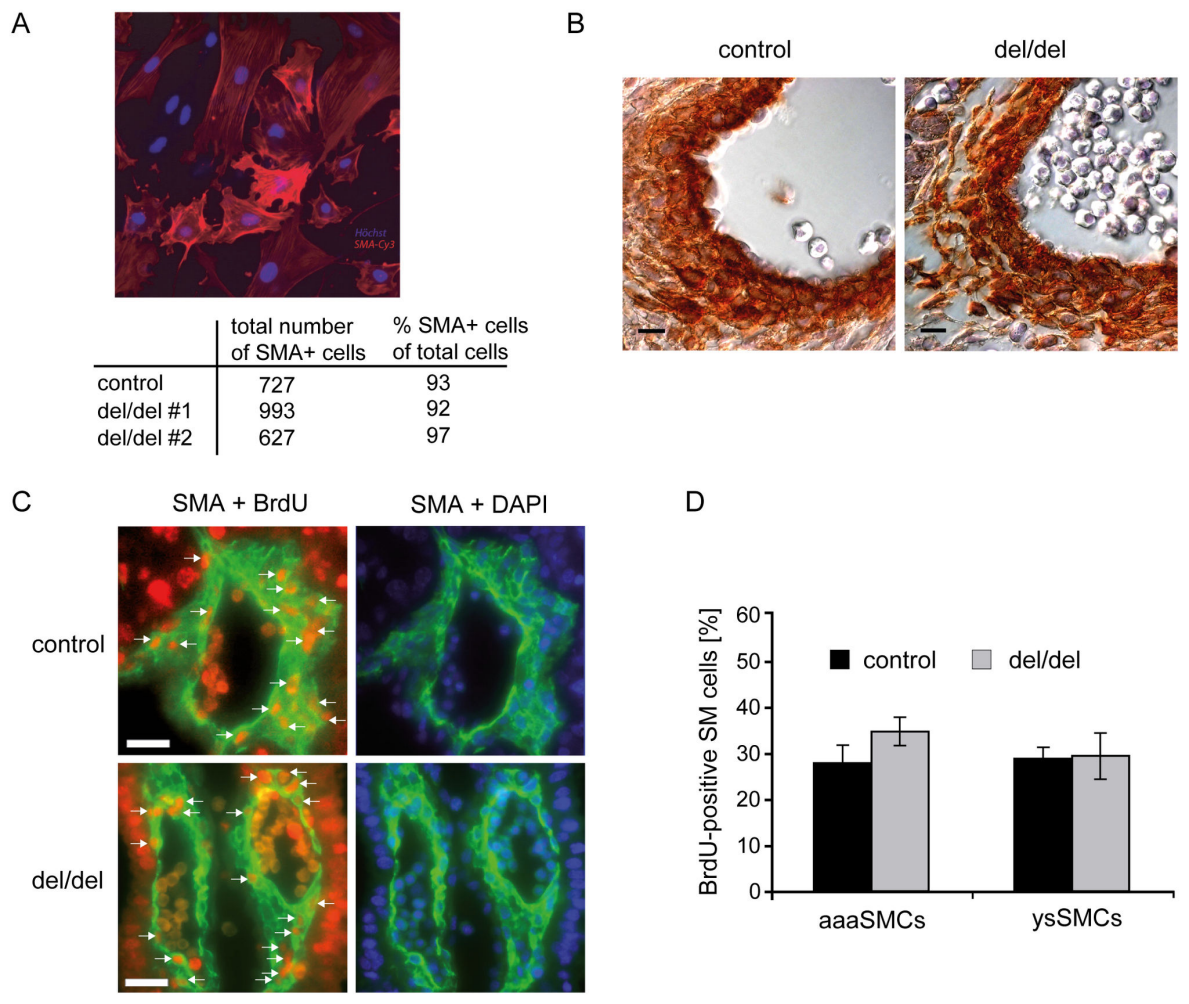
Regular differentiation, recruitment and proliferation of *Prdm6*-deficient smooth muscle cells. (**A**) ES cell lines were differentiated *in* *vitro* into SMA-positive (stained in red) smooth muscle-lineage cells (nuclei are stained in blue by DAPI). The numbers and frequencies of smooth muscle-lineage cells obtained from wild type control and two independent *Prdm6*-deficient (del/del) ES cell lines (#1 and #2) are given in the table. (**B**) Smooth muscle cells are normally recruited to aortic vessels of Prdm6^del/del^ embryos. E12.5 embryos, either Prdm6^wt/wt^ or Prdm6^del/del^, were immunohistochemically stained against SMA. Scale bars represent 10 µm. (**C**) Normal proliferation of SMCs in the yolk sac vasculature of *Prdm6* ^del/del^ embryos. Pregnant *Prdm6* ^wt/del^ mice from matings with *Prdm6* ^wt/del^ male mice were injected with BrdU at E11.5 and euthanized, and embryos with the wild type control genotype (wt/wt or wt/del) or *Prdm6*-deficient genotype (del/del) were sectioned and co-stained using antibodies to SMA (green fluorescence within the cytoplasm), BrdU (red fluorescent nuclei) and DAPI (blue, nuclear). Left: overlays of SMA and BrdU. Right: corresponding overlays of SMA and DAPI. Arrows indicate BrdU-positive SMCs. Representative yolk sac vessels are depicted. SMCs of the aortic arch arteries exhibited equivalent staining (not shown). Scale bars represent 20 µm. (**D**) Quantitative analysis of immunocytochemical staining, as shown in (C). Total SMCs (SMA + DAPI double-positive cells) and proliferating BrdU-positive SMCs (SMA + BrdU double-positive cells) were counted. The ratio of the latter to the former was defined as the proliferative index (percentage of BrdU-positive SM cells) for yolk sac SMCs (ysSMCs) and aortic arch artery SMCs (aaaSMCs). Data were obtained by counting at least 150 SMCs per vessel type from three embryos per genotype and are depicted as the mean +/- SD.

It was recently reported that Prdm6 might play a role in SMC function [3]. We therefore next tested whether *Prdm6* deficiency affects SMC differentiation and proliferation. To this end, we established *Prdm6*-deficient ES cell lines from the inner cell mass of early E3.5 *Prdm6* ^del/del^ embryos and differentiated these ES cells into defined vascular cell lineages under specific culture conditions *in vitro*. Of note, the *Prdm6* ^del/del^ ES cell lines differentiated regularly into smooth muscle alpha-actin (SMA)-expressing cells (i.e. pericytes or vascular SMCs [26]) at a rate and frequency comparable to wild type ES cell lines (Figure 3A), indicating that Prdm6 is dispensable for SMC-lineage differentiation. Moreover, *Prdm6* ^del/del^ ES cell lines also regularly differentiated into ECs and cardiomyocytes *in vitro* (data not shown). Immunohistochemical staining to SMA demonstrated that SMCs were regularly lining arterial vessel walls in *Prdm6* ^del/del^ embryos, thus indicating that recruitment of SMCs to the vasculature was intact (Figure 3B). To study the proliferation of embryonic vascular SMCs *in vivo*, we injected bromodeoxyuridine (BrdU) into pregnant mice and subsequently used immunohistology to determine the BrdU content within embryonic SMCs in different vascular regions. We did not observe significant differences in the frequencies of BrdU-positive SMCs between wild type control and *Prdm6*-deficient embryos, neither in SMCs in the aortic arch arteries nor in the yolk sac (Figure 3 C,D). In conclusion, Prdm6 does not seem to be required for either general SMC differentiation, recruitment to blood vessels or proliferation during embryonic development.

### SM22-Cre-induced *Prdm6* deletion results in perinatal death associated with pulmonary hemorrhage

**Figure 4:**
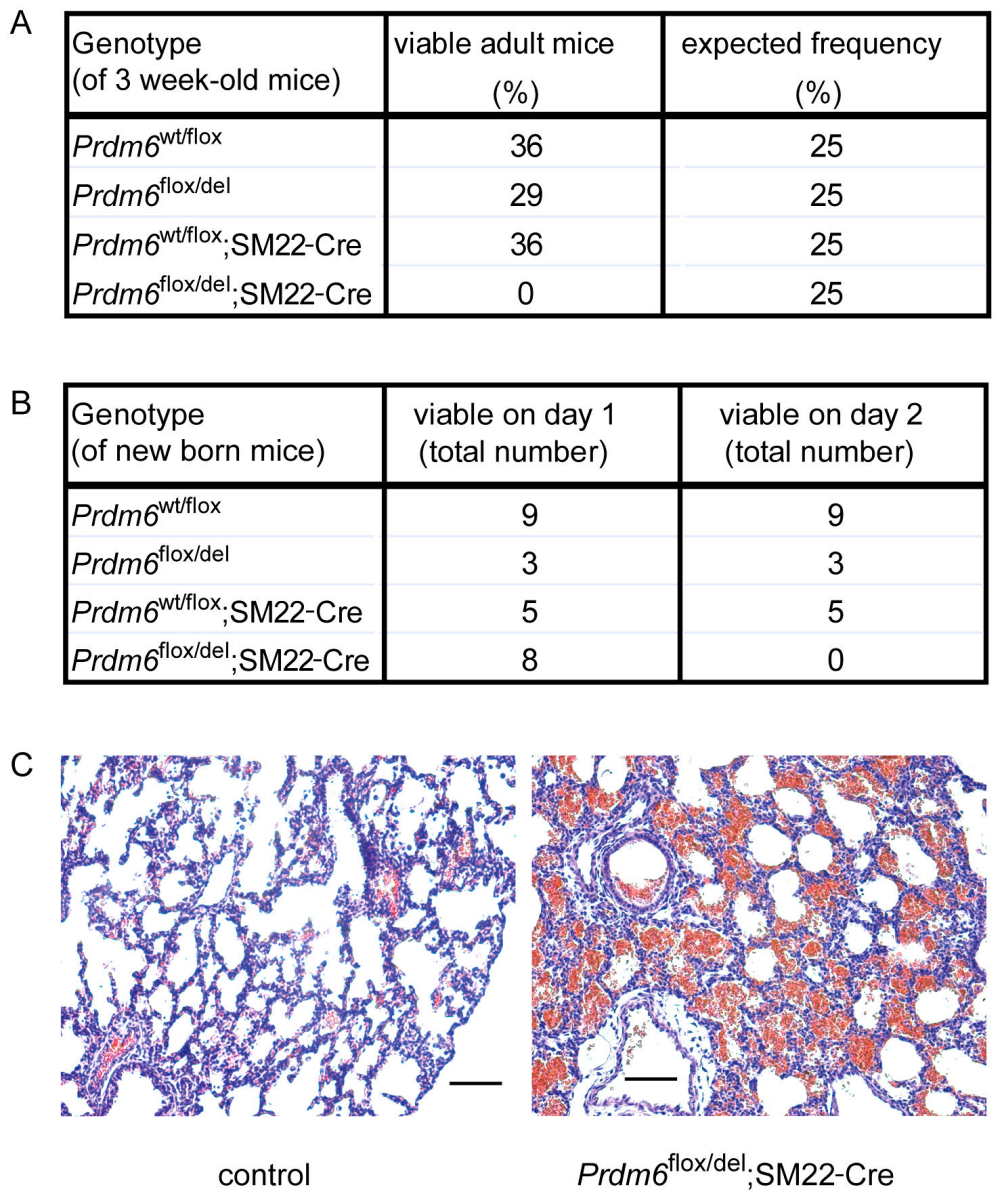
Selective disruption of *Prdm6* in vascular smooth muscle cells results in perinatal lethality. (**A**) *Prdm6* ^wt/del^;SM22-Cre mice were crossed with *Prdm6* ^flox/flox^ mice, and the genotypes of the offspring were analyzed at three weeks of age. The *Prdm6* ^flox/del^;SM22-Cre genotype leads to deletion of Prdm6 in the SMC lineage. The frequencies of the resulting genotypes were calculated from a total of 28 offspring animals and compared to the expected Mendelian frequencies. (**B**) Newborn mice from the same crosses as in (A) were observed at day 1 and day 2 after birth and subsequently were genotyped. (**C**) Lungs from newborn *Prdm6* ^flox/del^ control animals (viable) and SMC-conditional *Prdm6* ^flox/del^;SM22-Cre animals (deceasing) were embedded in paraffin, and sections were stained with hematoxylin and eosin. Scale bars correspond to 100 µm.

We crossed the *Prdm6* ^flox/del^ alleles into the SM22-Cre mouse line [27,28] to generate *Prdm6* ^flox/del^;SM22-Cre mice in which *Prdm6* is selectively disrupted in the SMC lineage . Although SMC-conditional *Prdm6* knockout mice were born at expected Mendelian frequency, we did not obtain viable adult SMC-conditional *Prdm6* knockout mice (Figure 4A) because all newborn *Prdm6* ^flox/del^;SM22-Cre pups died within 2 days after birth (Figure 4B). Interestingly, perinatal death induced by SM22-Cre-mediated conditional deletion of *Prdm6* was associated with massive hemorrhage in the lungs (Figure 4C). 

### Prdm6 regulates factors that are involved in angiogenesis

**Figure 5:**
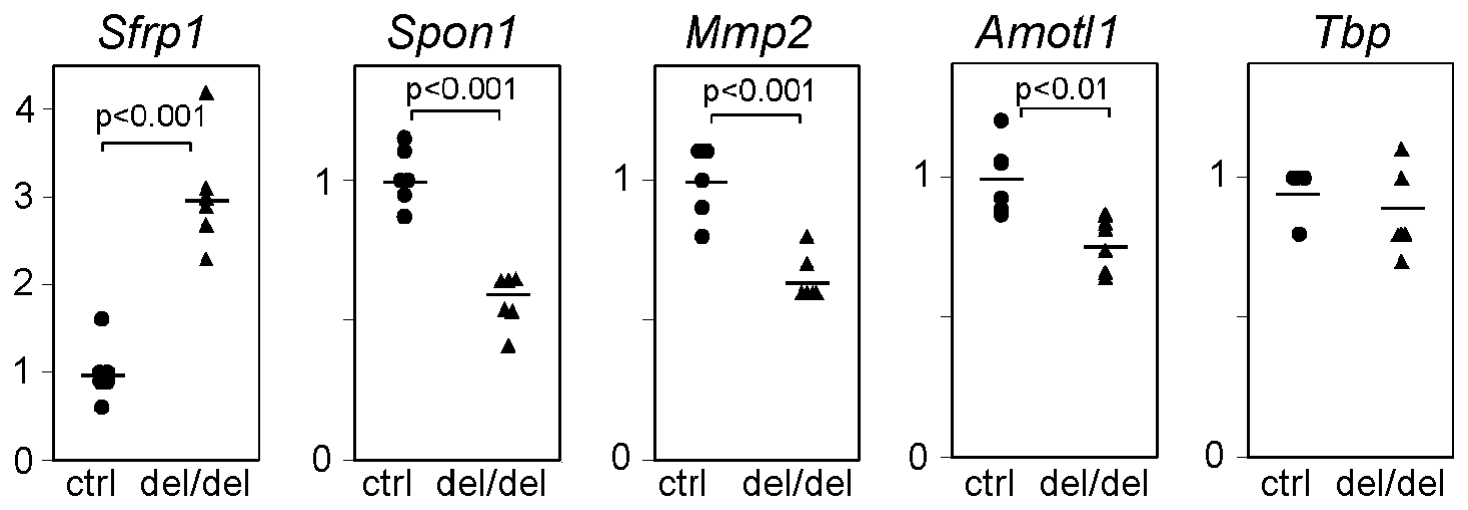
Deregulated expression of angiogenesis genes in *Prdm6*-deficient yolk sacs. Real-time RT-PCR analysis of selected transcripts identified *via* global gene expression profiling analysis (see Fig. S3). The expression values obtained from *Prdm6*-deficient (del/del) yolk sac samples were normalized to the expression values detected in wild type control samples. The housekeeping gene *Tbp* was expressed at equivalent levels in wild type and *Prdm6*-deficient yolk sacs.

Since Prdm6 acts as a transcription factor [3], we were interested in the identification of target genes that are physiologically controlled by Prdm6. Therefore, we performed genome-wide cDNA microarray analysis and compared gene expression patterns between wild type *Prdm6* ^wt/wt^ and knockout *Prdm6* ^del/del^ embryos. Because we observed an impact of Prdm6 deletion on vascular development, we compared the mRNA expression patterns in the yolk sacs of day E10.5 embryos, which are highly vascularized and easily accessible, allowing high quality RNA isolation. A total of 51 genes were found to be differentially expressed in *Prdm6* ^del/del^ yolk sacs compared to wild-type tissue (Figure S3). Only two genes (*Sfrp1* and *Mtap1b*) were upregulated, while all of the other deregulated genes displayed decreased expression levels in the absence of Prdm6. Several of the differentially regulated genes have been previously implicated in angiogenesis, such as those coding for the Wnt signaling inhibitor Sfrp1, the extracellular matrix protein F-Spondin, and the matrix metalloproteinase MMP2. Quantitative RT-PCR (qPCR) analysis of selected genes confirmed the microarray data (Figure 5 ), indicating that Prdm6 directly or indirectly controls the expression of a set of genes that are implicated in vascular development, and possibly also in other developmental processes.

## Discussion

In this study we reveal an essential role of Prdm6 for the development of the cardiovascular system. *Prdm6* total knockout embryos (*Prdm6* ^del/del^) die during development with an onset of about E10.5. At later stages, *Prdm6* ^del/del^ embryos display signs of cardiac insufficiency, i.e. edema and progressive heart defects. Anti-CD31 staining revealed that a vessel network is present in Prdm6-deficient yolk sacs, indicating that vasculogenesis is intact in *Prdm6* deficient yolk sacs. However, the small vessel network of Prdm6-deficient yolk sacs displayed an altered patterning with increased vascular diameters and smaller avascular space when compared to wild type yolk sacs. Prdm6 deficiency therefore apparently affects aspects of angiogenesis. Embryonic lethal phenotypes involving intact vasculogenesis, but impaired angiogenesis have been described for mice that are deficient in a variety of genes, such as *Fzd5* [29], *Notch1* [30,31], *Jagged1* [32], *Hey1/Hey2* [33], *Smoothened* [34], *Eph-B4* and *Ephrin-B2* [35], *Angiopoietin* [36], *Tie2* [37], *Smad5* [38], *Quaking* [39], *HIF2*alpha- [40], *VE-PTP* [41], *SCL/Tal-1* [42], and *PI3K p110-α* [43]. Inactivation of those key regulators of angiogenesis results in embryonic death latest by E11.0. Compared to that, *Prdm6* ^del/del^ embryos start to die around E10.5 with clearly reduced but countable numbers still alive and without clear morphological defects at E12.5. All *Prdm6* ^del/del^ embryos that can be identified at E12.5 however are edematous, anemic pale and obviously are deceasing. While the above mentioned gene knockouts of angiogenic key regulators arrest angiogenesis in the yolk sac already at the level of the primitive primary plexus with defective development of the large vessel system, *Prdm6* ^del/del^ yolk sacs do possess large vessels and the observed angiogenesis defect of the small vessel network in *Prdm6* ^del/del^ yolk sacs is rather mild. Thus, it is questionable whether the observed subtle changes in vascular patterning can be responsible for the embryonic lethal phenotype of the Prdm6^del/del^ embryos. It appears likely that *Prdm6*-deficiency might directly induce the observed heart defect that might be the primary cause of embryonic death. Further studies are required to resolve this issue.

Davis et al. proposed that Prdm6 is a transcriptional repressor that suppresses SMC differentiation and promotes SM proliferation [3]. We therefore investigated SMCs in *Prdm6* ^del/del^ total knockout embryos in order to test whether *Prdm6* deficiency might have an effect on SMCs that potentially could contribute to the cardiovascular phenotype. However, we could not detect defects in the overall capacity of *Prdm6*-deficient SMCs to differentiate, proliferate and to be recruited to blood vessels *in vivo*. Moreover, *Prdm6*-deficient ES cells were able to differentiate into the pericyte lineage *in vitro*, to the same extent as wild type ES cells. These findings indicate that Prdm6 function might be required for alternative aspects of SMC function or in additional cell types during vascular development, e.g. the endothelial lineage as has been suggested by Wu et al. [21]. Future experiments are required to address these questions.

Even though we did not detect SMC defects in *Prdm6* ^del/del^ total knockout embryos, we crossed our floxed *Prdm6* allele to a SM22-Cre deleter line which induces Cre-mediated recombination in the SMC lineage [27] but also in other selected cell types such as mesothelial cells in the yolk sac and in cardiomyocytes during early heart development [44-46]. Conditional deletion of *Prdm6* by the SM22-Cre driver did not result in embryonic death, which is an additional indication that *Prdm6*-deficiency in SMCs might not be the main cause for the defect in embryonic development as we observe it in *Prdm6* ^del/del^ total knockout embryos. For the same reason, it is unlikely that Prdm6 plays essential roles in mesothelial cells or cardiomyocytes during early stages of embryonic development. Interestingly however, SM22-Cre driven conditional deletion of *Prdm6* resulted in postnatal death associated with lung hemorrhage. In lung, the expression of SM22-Cre has been demonstrated to be confined to vascular smooth muscle cells [27]. Thus, it might be assumed that smooth muscle cells require *Prdm6* for maintaining pulmonary vessel integrity. Alternatively, however, it cannot be ruled out that SM22-driven deletion of *Prdm6* in cardiomyocytes during early development [45] eventually causes heart failure in newborn mice with subsequent secondary blood congestion and hemorrhage in the lungs. This aspect needs to be addressed by future studies. The cardiovascular phenotype that we observe in the *Prdm6* ^del/del^ total knockout embryos and pulmonary hemorrhage that we observe in the SM22-Cre driven conditional deletion of *Prdm6* are in line with the reported physiological expression of *Prdm6* within the vascular system [3,21]. However, further additional analyses will be necessary to clarify which cell types and which molecular mechanisms are contributing to the cardiovascular defects after total Prdm6 disruption during development or after conditional inactivation of Prdm6 by SM22-Cre.

Prdm6 is a transcription factor that can associate with chromatin-remodeling enzymes, such as heterochromatin protein-1 (HP1-β), histone deacetylases HDAC1, - 2, and -3, the histone acetyltransferase p300, and the histone methyl transferase G9a, to modulate gene expression [3]. In our microarray analysis, we identified approximately 50 significantly deregulated transcripts, among which only two genes were upregulated, while all of the others were downregulated in *Prdm6*-deficient yolk sacs. Considering that Prdm6 was assumed to function as a transcriptional repressor [3], we actually expected more transcripts to be upregulated rather than downregulated in the absence of Prdm6. However, also the related Prdm5 protein was reported to possess the capacity to mediate both negative and positive transcriptional regulation, presumably depending on its interaction with additional transcriptional co-factors [47]. Our microarray data indicate that Prdm6 could also act as both a positive and negative regulator of transcription. Interestingly, among direct or indirect Prdm6 targets, we recognized six genes that were previously associated with angiogenic processes: Sf*rp1* [48], *Spon1* [49], *Rhob* [50,51], *Mmp2* [52,53], *Arrb1* [54], and *Amotl1* [55]. The upregulation of *Sfrp1* in the *Prdm6*-deficient embryos might be of special interest, as Sfrp1 acts as an antagonist of the Wnt/frizzled pathway expressed in smooth muscle cells [56]. Moreover, Wnt/frizzled signaling plays a critical role in distinct steps of embryonic vascular development [57]. For example, disruption of *Frizzled-5* leads to embryonic lethality by E11.5, accompanied by defects in yolk sac angiogenesis [29]. Moreover, deficiency of *Wnt7b* results in postnatal death due to lung hemorrhage caused by vascular leakage and subsequent respiratory failure [58], similar to what we observed upon selective Prdm6 deletion in SMCs using SM22-Cre. As an independent study has additionally revealed that Prdm6 can directly regulate Wnt4 expression [3], we speculate that Prdm6 might modulate angiogenesis partly through effects on the Wnt/frizzled pathway. Further mechanistic studies are required to test this hypothesis and to understand which target genes are directly or indirectly regulated by Prdm6, thereby also providing hints concerning a potential role of PRDM6 in lymphomagenesis. The present study is a starting point for future investigations of PRDM6 *in vivo* functions with our conditional knockout mouse model being a valuable tool to further define the role of PRDM6 in the cardiovascular system by its selective deletion in e.g. the endothelial lineage or in cardiomyocytes and to study the possible impact of Prdm6 in other physiological processes, such as neurogenesis with which Prdm6 expression has been reported to be associated [22].

## Materials and Methods

### Ethics Statement

All animal work was conducted in accordance with German Federal Animal Protection Laws and approved by the Institutional Animal Care and Use Committee at the Technical University of Munich.

### Generation of *Prdm6* conditional knockout mice and flp/Cre deleter strains

Exon 3 of *Prdm6* was flanked by *loxP* sites *via* homologous recombination in E14K ES cells according to standard procedures [59]. The embryonic stem (ES) cells containing the correctly recombined (rec) *Prdm6* locus (Prdm6^wt/rec^) still also contained the FRT-flanked neomycin resistance selection cassette. Standard ES cell technologies were used to generate germline mutant *Prdm6* ^wt/rec^ mice. Crossing with flp recombinase deleter mice [60] resulted in deletion of the neomycin resistance cassette and produced *Prdm6* ^wt/flox^ mice. The following mouse strains were used: flp deleter mice (Jax human ß-actin FLPe deleter strain B6;SJL-Tg(ACTFLPe)9205Dym/J), Cre deleter mice (Jax human CMV-Cre deleter strain B6.C-Tg(CMV-cre)1Cgn/J), and SM22-Cre (i.e. SM22alpha-Cre) mice (Jax Tg(Tagln-Cre)1Her/J). The mice were housed in a specific pathogen-free facility according to FELASA recommendations (http://www.felasa.eu). Littermates were used in all experiments. 

### Genotyping PCR, RT-PCR and qPCR

For genotyping of the *Prdm6* wt, del and flox alleles, the following primer combinations were used. wt allele: fwd: 5'-agacagaacatcaagaagggtag-3' plus wt rev: 5'-ggcctcctgggaactgattag-3' (260 bp band); del allele: fwd: 5'-agacagaacatcaagaagggtag-3' plus del rev: 5'-ccagatttgtgcaccctttaagc-3' (570 bp); and flox allele: 5'-agacagaacatcaagaagggtag-3' plus flox rev: 5'-gatatcgctagcgggaagttc-3' (380 bp). RT-PCR and qPCR were performed as previously described [61]. The following primer pairs were used: *Prdm6* exon 3 wt allel specific primers (5’-taacagtagttcagtacaggtcg-3’ plus 5’-aagagggagaaattcctgctg-3’), *Prdm6* knockout (del) allele specific primers detecting alternative exon2-exon4-splicing (fwd: 5’-gcatctctgggaggtcgaat-3’ plus rev: 5’-ggtggaagggacgttcaagt-3’). To perform real-time quantitative PCR, the following primer pairs were designed to span exon-exon boundaries: *Sfrp1* (5'-cctgaggactccactttatagccta-3' plus 5'- ggaatcactattaacatacgtgataacatc-3'), *Spon1* (5'-tactcatgcatctgttaaagctacca-3' plus 5'- gttgtacatagatgtggctggacata-3'), *Mmp2* (5'-gtgttcttcgcagggaatgagta-3' plus 5'- cacttcattgtatctccagaacttgtct-3'), *Amotl1* (5'-ccagcggactctggtatcca-3' plus 5'- ggctgaccaacagtatccatattca-3') and *Tbp* (5'-ccaccagcagttcagtagctatga-3' plus 5'-tgctctaactttagcacctgttaatacaac-3'). 

### Southern blot and Northern blot analyses

Southern and Northern hybridizations were performed according to standard protocols [62,63]. For embryonic expression analysis, a Mouse Embryo Full Stage Blot (Seegene, Seoul, Korea) was used. 

### Histology, Immunohistochemistry, and BrdU incorporation assays

To obtain histological sections and perform H&E staining, standard protocols were used, as described previously [64]. For anti-CD31 staining, primary rat anti-mouse CD31 Ab (BD Pharmingen, clone MEC 13.3) was used, followed by fluorescent labeling with a secondary anti-rat Cy2 Ab or anti-rat Cy3 Ab (both from Jackson Immuno Research). To determine the proliferative index in smooth muscle cells of embryos and yolk sacs, pregnant mice were injected intraperitoneally three times every 2 h with 1.5 mg BrdU in 150 µl PBS. Then, the mice were euthanized, and the embryos were sectioned. Epitope retrieval was achieved through boiling in citrate buffer (10 mM citrate, pH 6.0 + 0.05% Tween-20) for 20 min and a subsequent DNase I digestion (DNase I, Roche, grade 2, 1 mg/ml in PBS-Tween) for 1 h at 37°C. The sections were double-stained with a rat anti-BrdU Ab (1:50 in PBS-Tween, Serotec) and mouse anti-SMA Ab (1:500, Sigma clone 1A4) and incubated with the secondary antibodies anti-rat Cy3 (Jackson ImmunoResearch) and anti-mouse IgG2a FITC (Southern Biotech). The samples were analyzed with a Zeiss Axioplan 2 fluorescence microscope.

### ES cell generation and differentiation assays

ES cell clones were obtained according to standard protocols [65], with the addition of the MEK1 inhibitor PD98059 (50 µM, NEB). Differentiation assays were performed as described previously [66]. Briefly, ES cells were co-cultured with OP 9 cells in differentiation medium containing 10% FCS (PAN Biotech) and 10^-4^ M beta-mercaptoethanol (Sigma-Aldrich) in alpha-MEM (Gibco/Invitrogen) and then FACS sorted to detect Flk1-positive, Cadherin-negative lateral plate mesodermal cells (Flk1 Ab: clone AVAS12, eBioscience; Cadherin Ab, clone ECCD2). To achieve mural cell differentiation, 2x10^4^ sorted mesodermal cells were cultivated for 4 days on Collagen IV-coated plates. The cells were fixed in methanol containing 5% DMSO and stained with a monoclonal anti-SMA-Cy3 Ab (Sigma-Aldrich, Clone 1A4).

### cDNA microarray analysis

E10.5 yolk sacs from six *Prdm6* ^del/del^ and six wild type control mice were dissected on ice in DEPC-treated PBS, shock frozen in liquid nitrogen and stored at -80°C. Total RNA was isolated using RNeasy Mini kits (Qiagen), and 400 ng of RNA was amplified according to the instructions of the Target AMP^TM^ 1-Round aRNA Amplification Kit 103 (Epicentre Biotechnologies). Genome-wide cDNA microarrays were generated, hybridized and analyzed as described recently [67]. The selection of significantly differentially expressed genes showing reproducible up- or down-regulation included less than 5% false positives (FDR) in combination with fold changes of >1.3. The expression data were submitted to the GEO database (GSE9065), where a full description of our microarray results is also available (GPL4937).

## Supporting Information

Figure S1 **Generation of a conditional *Prdm6* allele.** (**A**) Amino acid sequence of the murine Prdm6 protein according to GenBank accession number NP_001028453. Two methionine start residues are indicated by circles: the first corresponds to the sequence proposed by Wu et al. [21], the second was described by Davis et. al [3] . The PR domain in the central part of the sequence is indicated in bold, whereas the zinc finger region is underlined. Exon-exon borders are marked with dashed vertical lines, and exon numbers are given to indicate by which exons the different parts of the protein are encoded. (**B**) Targeting strategy for homologous recombination at the *Prdm6* locus. The region containing exon 3 of the Prdm6 wt locus, the targeting vector and the distinct recombinant alleles (rec, flox, del) are shown. The restriction fragment lengths produced by Afl II digestion are indicated for the various wt and recombinant alleles. The homology arms for recombination are drawn as strong lines and the probe region used for Southern blot analysis is indicated. LoxP sites are represented by triangles, the neomycin selection cassette by NEO and the FRT sites by closed circles. Homologous recombination in ES cells produced the recombinant (rec) locus containing the *LoxP*-flanked exon 3 region and the FRT-flanked NEO cassette. ES cells carrying the rec locus were then transferred into the germ line of mice. Crossing mice with the rec locus with flp deleter mice resulted in deletion of the NEO cassette and generated the flox allele. Crossing flox mice with Cre deleter mice resulted in the deletion of exon 3, thus generating the del allele. (**C**) Southern blot analysis of genomic DNA from thymi of mice with the respective genotypes demonstrated the presence of the expected allele sizes, as defined in (B). Genomic DNA was digested with AflII and hybridized with the probe as depicted in (B). (**D**) RT PCR analysis using cDNA from yolk sacs of the indicated genotypes as templates. The wild type *Prdm6* transcript was amplified using a forward primer that specifically binds to the *Prdm6* exon 2-exon 3 splice fusion site and therefore cannot anneal to the exon 3-deficient knockout (del) allele. The *Prdm6* knockout (del) transcript was amplified using a forward primer that specifically binds to the alternatively spliced exon2-exon 4 fusion site that is only present in the knockout (del) allele but not in the wild type allele.
(PDF)

Figure S2 **Quantitative morphometric analysis of the yolk sac vasculature.** The vascular networks of a representative wild type control (A) and a *Prdm6* knockout (B) yolk sac were analyzed by measuring the avascular space (i.e. intercapillary space) and mean vessel diameters. The left panel shows the original image of the anti-CD31 stains of whole mount yolk sacs. The white areas in the center panels indicate the avascular spaces as measured by the histogram function of the Photoshop CS6 software. The right panels indicate all points where vessel diameters were measured using the ruler function of Photoshop CS6 software. Vessel diameters were determined in between all branching points. Scale bars correspond to 200 µm.
(PDF)

Figure S3 **Heat plot of gene expression profiles from yolk sacs of *Prdm6*-deficient embryos.** One dye-flip pair represents two experimental replicates of each of the six analyzed E10.5 yolk sacs. Official gene symbols are given. The scale bar indicates the mean ratio of fold induction. Red indicates upregulated and green downregulated genes in *Prdm6* ^del/del^ yolk sacs compared to wild type control yolk sacs.
(PDF)
