# Supplementary material for: Prdm6 Is Essential for Cardiovascular Development In Vivo
Source: PLoS One. 2013 Nov 21;8(11):e81833. doi: 10.1371/journal.pone.0081833 (PMC3836774; doi:10.1371/journal.pone.0081833)
Supplement: Figure S1 — Generation of a conditional Prdm6 allele. (A) Amino acid sequence of the murine Prdm6 protein according to GenBank accession number NP_001028453. Two methionine start residues are indicated by circles: the first corresponds to the sequence proposed by Wu et al. [21], the second was described by Davis et. al [3] . The PR domain in the central part of the sequence is indicated in bold, whereas the zinc finger region is underlined. Exon-exon borders are marked with dashed vertical lines, and exon numbers are given to indicate by which exons the different parts of the protein are encoded. (B) Targeting strategy for homologous recombination at the Prdm6 locus. The region containing exon 3 of the Prdm6 wt locus, the targeting vector and the distinct recombinant alleles (rec, flox, del) are shown. The restriction fragment lengths produced by Afl II digestion are indicated for the various wt and recombinant alleles. The homology arms for recombination are drawn as strong lines and the probe region used for Southern blot analysis is indicated. LoxP sites are represented by triangles, the neomycin selection cassette by NEO and the FRT sites by closed circles. Homologous recombination in ES cells produced the recombinant (rec) locus containing the LoxP-flanked exon 3 region and the FRT-flanked NEO cassette. ES cells carrying the rec locus were then transferred into the germ line of mice. Crossing mice with the rec locus with flp deleter mice resulted in deletion of the NEO cassette and generated the flox allele. Crossing flox mice with Cre deleter mice resulted in the deletion of exon 3, thus generating the del allele. (C) Southern blot analysis of genomic DNA from thymi of mice with the respective genotypes demonstrated the presence of the expected allele sizes, as defined in (B). Genomic DNA was digested with AflII and hybridized with the probe as depicted in (B). (D) RT PCR analysis using cDNA from yolk sacs of the indicated genotypes as templates. The wild type Prdm6 trans [file pone.0081833.s001.pdf]

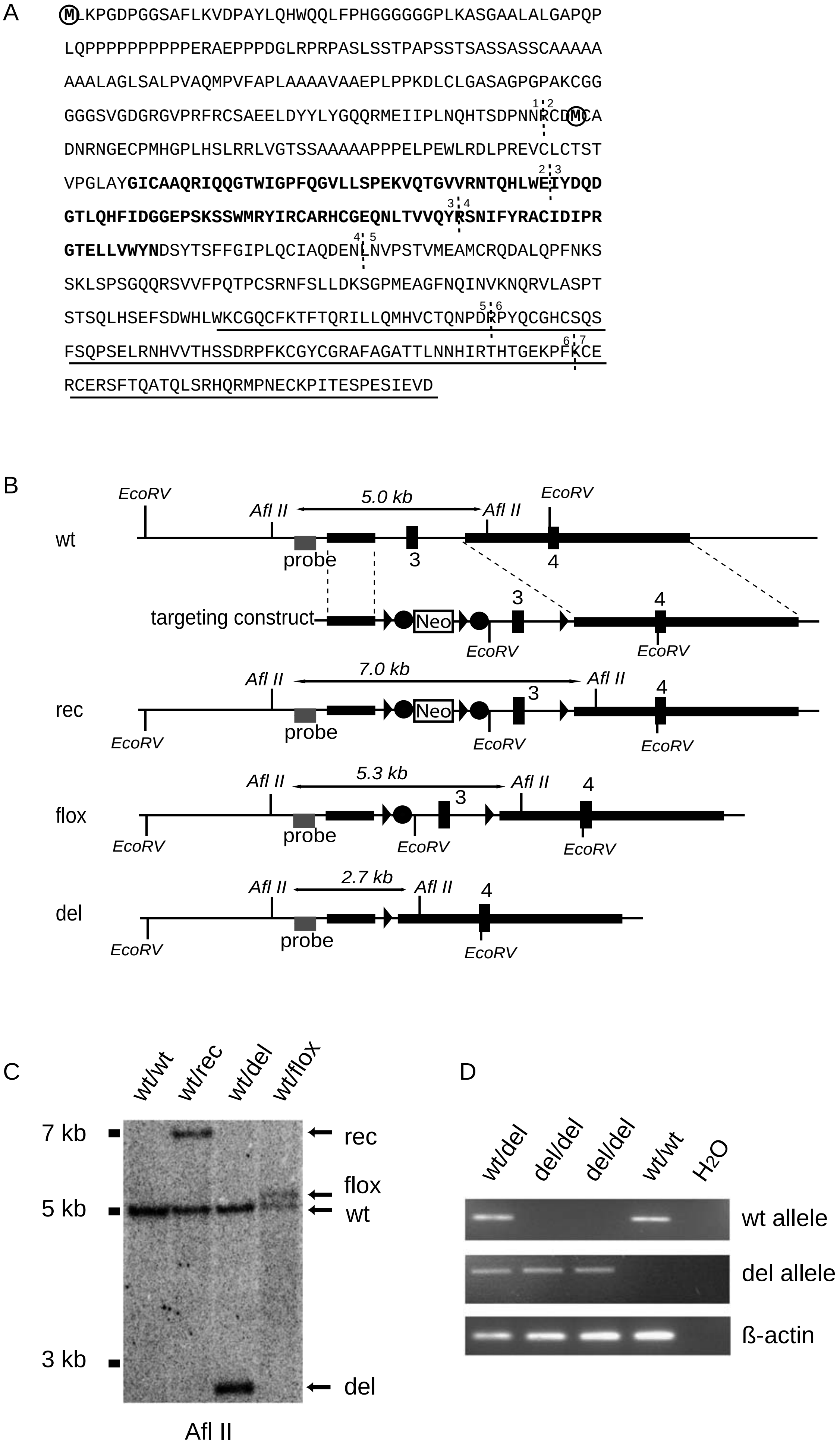
A
MLKPGDPGGSAFLKVDPAYLQHWQQLFPHGGGGGGPLKASGAALALGAPQP
LQPPPPPPPPPPERAEPPPDGLRPRPASLSSTPAPSSTSASSASSCAAAAA
AAALAGLSALPVAQMPVFAPLAAAAVAAEPLPPKDLCLGASAGPGPAKCGG
1 2
GGGSVGDGRGVPRFRCSAEELDYYLYGQQRMEIIPLNQHTSDPNNRCDMCA
DNRNGECPMHGPLHSLRRLVGTSSAAAAAPPPELPEWLRDLPREVCLCTST
2 3
VPGLAYGICAAQRIQQGTWIGPFQGVLLSPEKVQTGVVRNTQHLWEIYDQD
3 4
GTLQHFIDGGEPSKSSWMRYIRCARHCGEQNLTVVQYRSNIFYRACIDIPR
4 5
GTELLVWYNDSYTSFFGIPLQCIAQDENLNVPSTVMEAMCRQDALQPFNKS
SKLSPSGQQRSVVFPQTPCSRNFSLLDKSGPMEAGFNQINVKNQRVLASPT
5 6
STSQLHSEFSDWHLWKCGQCFKTFTQRILLQMHVCTQNPDRPYQCGHCSQS
6 7
FSQPSELRNHVVTHSSDRPFKCGYCGRAFAGATTLNNHIRTHTGEKPFKCE
RCERSFTQATQLSRHQRMPNECKPITESPESIEVD
B
EcoRV
Afl II
5.0 kb
Afl II
EcoRV
wt
probe
3
4
3
4
targeting construct
Neo
EcoRV
EcoRV
Afl II
7.0 kb
Afl II
4
3
rec
Neo
probe
EcoRV
EcoRV
EcoRV
Afl II
5.3 kb
Afl II
4
3
flox
probe
EcoRV
EcoRV
EcoRV
Afl II
2.7 kb
Afl II
4
del
probe
EcoRV
EcoRV
C
wt/wt
wt/rec
wt/del
wt/flox
7 kb
rec
flox
5 kb
wt
3 kb
del
Afl II
D
wt/del
del/del
del/del
wt/wt
H_2O
wt allele
del allele
ß-actin
