# Supplementary material for: Prdm6 Is Essential for Cardiovascular Development In Vivo
Source: PLoS One. 2013 Nov 21;8(11):e81833. doi: 10.1371/journal.pone.0081833 (PMC3836774; doi:10.1371/journal.pone.0081833)
Supplement: Figure S2 — Quantitative morphometric analysis of the yolk sac vasculature. The vascular networks of a representative wild type control (A) and a Prdm6 knockout (B) yolk sac were analyzed by measuring the avascular space (i.e. intercapillary space) and mean vessel diameters. The left panel shows the original image of the anti-CD31 stains of whole mount yolk sacs. The white areas in the center panels indicate the avascular spaces as measured by the histogram function of the Photoshop CS6 software. The right panels indicate all points where vessel diameters were measured using the ruler function of Photoshop CS6 software. Vessel diameters were determined in between all branching points. Scale bars correspond to 200 µm. (PDF) [file pone.0081833.s002.pdf]

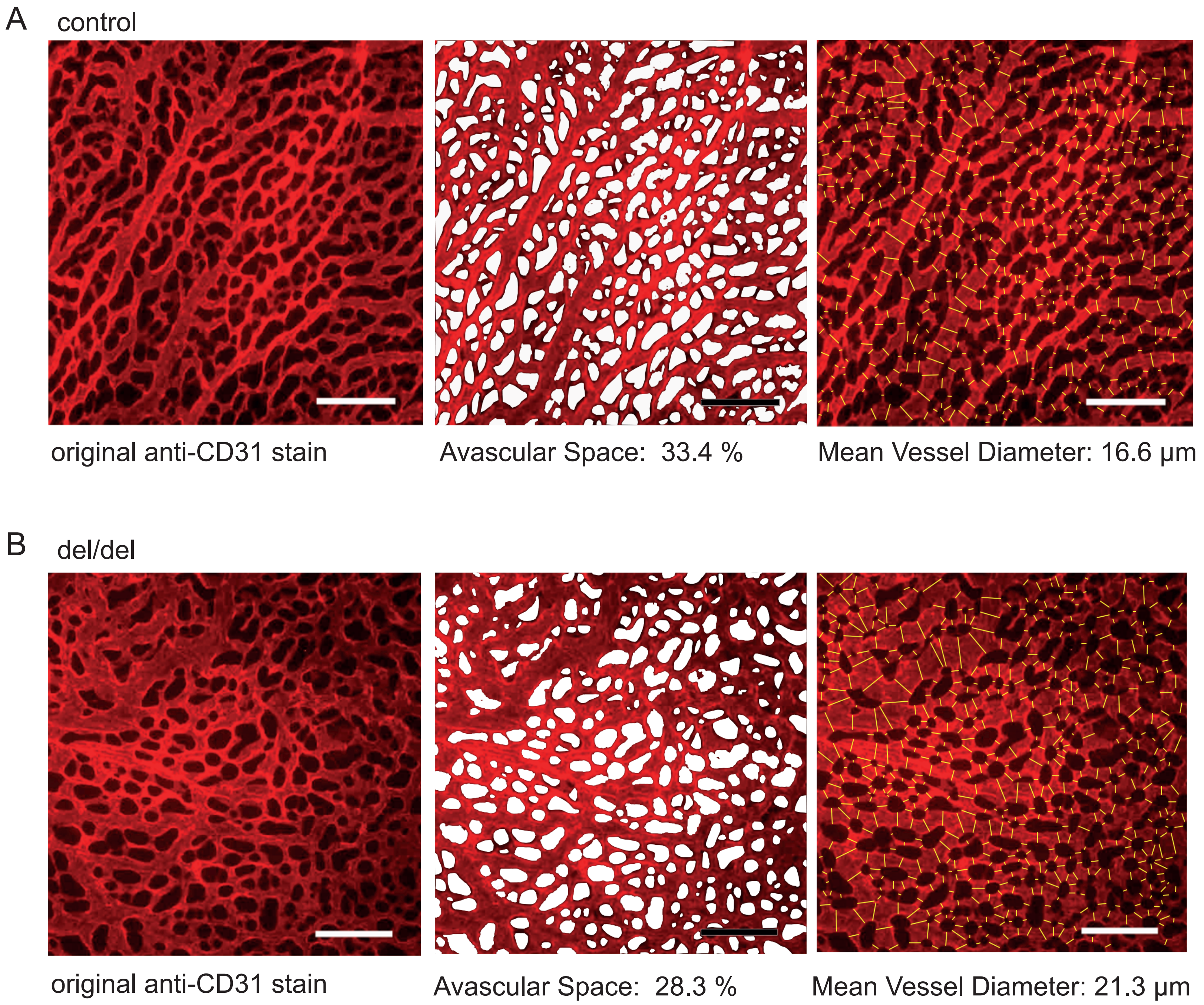
A
control
original anti-CD31 stain
Avascular Space: 33.4 %
Mean Vessel Diameter: 16.6 µm
B
del/del
original anti-CD31 stain
Avascular Space: 28.3 %
Mean Vessel Diameter: 21.3 µm
