# Supplementary material for: Prdm6 Is Essential for Cardiovascular Development In Vivo
Source: PLoS One. 2013 Nov 21;8(11):e81833. doi: 10.1371/journal.pone.0081833 (PMC3836774; doi:10.1371/journal.pone.0081833)
Supplement: Figure S3 — Heat plot of gene expression profiles from yolk sacs of Prdm6-deficient embryos. One dye-flip pair represents two experimental replicates of each of the six analyzed E10.5 yolk sacs. Official gene symbols are given. The scale bar indicates the mean ratio of fold induction. Red indicates upregulated and green downregulated genes in Prdm6 del/del yolk sacs compared to wild type control yolk sacs. (PDF) [file pone.0081833.s003.pdf]

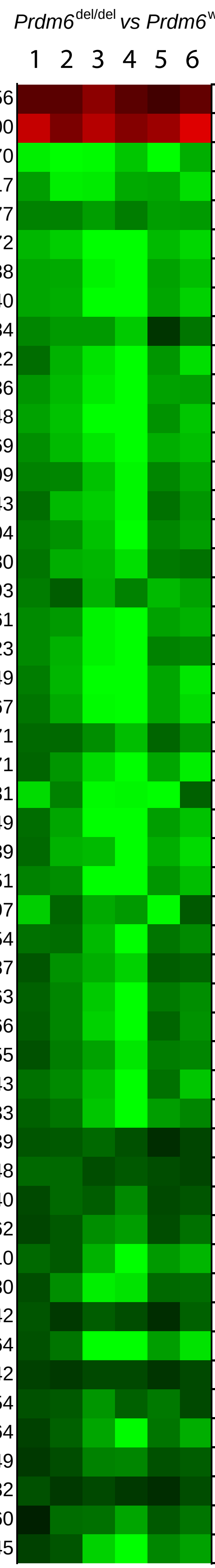

Mean ratio	Array TAG ID	Gene symbol	Comment
1,56	MG-6-30j4	Mtap1b	Microtubule-associated protein 1 B
2,00	MG-16-171f10	Sfrp1	Secreted frizzled-related sequence protein 1
-2,70	MG-8-117j20	Man2b2	mannosidase 2, alpha B2
-2,17	MG-8-73p2	Spon1	Spondin 1, (f-spondin) extracellular matrix protein
-1,77	MG-4-6h18	Ifitm3	Interferon induced transmembrane protein 3
-2,72	MG-14-64n9	Mark3	MAP/microtubule affinity-regulating kinase 3
-2,38	MG-15-214i1	Rhob	Ras homolog gene family, member B
-2,40	MG-3-97p3	Hemt1	hematopoietic cell transcript 1
-1,84	MG-8-29b14	Dctn2	Dynactin 2
-2,22	MG-4-3e6	Alas2	Aminolevulinic acid synthase 2, erythroid
-2,36	MG-8-55p15	Iqcf4	IQ motif containing F4
-2,48	MG-4-146n10	Plcl2	Phospholipase C-like 2
-2,69	MG-4-145f7	Scl43a2	solute carrier family 43, member 2
-2,09	MG-3-16e23	Slc9a9	Solute carrier family 9, isoform 9
-2,43	Fbxo32	Fbxo32	F-box only protein 32,3
-2,04	MG-8-71j2	Nup155	Nucleoporin 155
-1,80	MG-4-146i20	Clptm1	Cleft lip and palate associated transmembrane protein 1
-1,93	MG-3-18a15	CR521370	
-2,61	MG-4-3b2	Hba-a2	hemoglobin alpha, adult chain 2
-2,23	MG-4-4k23	E030041M21Rik	
-2,49	MG-68-143l7	Hbb-b2	hemoglobin, beta adult minor chain
-2,67	MG-4-3k1	Hba-a1	Hemoglobin alpha, adult chain 1
-1,71	MG-8-40g12	Rad9	RAD9 homolog
-2,71	MG-47-1h17	Hbb-bh1	Hemoglobin Z, beta-like embryonic chain
-2,31	MG-15-3b23	Col11a1	Procollagen, type XI, alpha 1
-2,49	MG-8-42b9	H3f3a	H3 histone, family 3A
-2,39	MG-8-11g1	Slc4a1	Solute carrier family 4 (anion exchanger), member 1
-2,51	MG-4-6d16	Tap1	Transporter 1, ATP-binding cassette, sub-family B
-1,97	Hoxc8	Hoxc8	Homeo box C8
-2,54	MG-4-4h13	Grrp1	glycine/arginine rich protein 1
-1,87	MG-8-117l6	Rpl11	Ribosomal protein L11
-2,63	MG-4-86f7	Hb2-a2	hemoglobin alpha, adult chain 2
-2,66	MG-4-147o3	Hba-a1	Hemoglobin alpha, adult chain 1
-2,55	MG-4-5j21	Il6st	Interleukin 6 signal transducer
-2,43	MG-8-86g2	1110017I16Rik	
-2,33	MG-8-40d4	Hba-a1	Hemoglobin alpha, adult chain 1
-1,39	MG-15-2j1	Mmp2	Matrix metalloproteinase 2
-1,48	MG-4-5e2	Ugt1a6	UDP glycosyltransferase 1 family, polypeptide A6
-1,40	MG-8-13e2	Xrn1	5'-3' exoribonuclease 1
-1,62	MG-3-10n5	Nudt4	Nudix (nucleoside diphosphate linked moiety X)-type motif 4
-2,10	MG-8-84a9	Eraf	Erythroid associated factor
-2,80	MG-4-2e20	Hba-a1	Hemoglobin alpha, adult chain 1
-1,42	MG-16-108a19	Mbnl1	Muscleblind-like 1
-2,64	MG-4-146d10	Tysnd1	Trypsin domain containing 1
-1,42	MG-8-16n9	Arrb1	Arrestin, beta 1
-1,54	MG-8-96d13	2210411K11Rik	
-2,64	MG-4-3k8	1300018I05Rik	
-1,49	MG-4-148g1	Hist2h3c2	Histone 2, H3c2
-1,32	MG-3-3f8	Amotl1	Angiomotin-like 1
-1,60	MG-3-218p12	Car2	Carbonic anhydrase 2
-2,45	MG-4-5m17	Hba-a2	hemoglobin alpha, adult chain 2
